# Foreign Correspondence

**Published:** 1870-02

**Authors:** 


### Foreign Correspondence.

R. R. ALLGEMEINEN KRAUKENHAUS,

*Vienna, Dec. 11th, 1869.*

DEAR EXAMINER:—Dr. Truehart, of Virginia, formerly of Galveston, Texas, has introduced some new instruments and modifications of instruments into obstetric practice, during his study here, of which I will only give a cursory notice, at this time, since the final form of all of them is not yet determined; and when so, the Doctor will probably lay a description of his instruments and their practicality before the profession. And, first, I may mention his decapitator, to be used in place of the blunt-hook. The last form of it I have seen consisted of a silk cord, wound with wire, and about three feet long, having, at either end, a loop, admitting the point of the index-finger, and thus carried around the child's neck. A strong wire or steel rod, having a hook at one end, is then introduced hooked into the loop and drawn out. Both ends of the wire are now slipped through a cylinder or tube, which is pressed firmly against the child's neck. The wire is then worked similar to a chain saw, and the neck divided. His next instrument is a modification of the perforator, and another I may call an *extractor*, to be used in place of the *cranio-clasp.* It consists of a piece of steel, about three inches long and an inch wide, slightly curved, to the middle of which an iron rod and handle are attached, by means of a hinge or joint. After the perforation has been made, the curved piece is introduced, parallel to the rod, and made to assume a cron position, thus extending an inch or more each side of the perforation, on the inside of the cranium. The extraction is then made. In one of the handles of the obstetric forceps, he has placed a screw, with an oval head, which fits into a socket of the other handle, and thus preventing too great pressure on the child's head, by a too close approximation of the handle. When the head is small or has become compressed, the screw can be turned up and the handles brought together. The screw also tends to keep the handles in place and prevents the blades working back and forth on the head. They are also made looser in the joint, which allows them to adapt themselves more perfectly to the head. A pair of these forceps has been sent to New York, for trial, and your readers will probably be made acquainted with the result. He has also introduced something of a new method for the resuscitation of stillborn children, which he has practised in Professor Braun's wards with excellent success.

By virtue of his inventions, etc., he has been allowed extra advantages in the obstetric wards and invited to present his instruments before one of the medical societies of the city.

They promise to supply a want of the profession.

Some time since, in one of my letters, I made mention of a case in Bonn, which was operated on for artificial anus, and with apparent success. I have now occasion to note another case, with contrary results. On the night of November 26th, a female child was born (three weeks premature), with no anal opening. An hour or two afterwards, Professor Bilroth was called and made an operation, but failed to find the rectum. The next morning, the infant was presented at the clinic, vomiting, cyanotic, and breathing irregularly. A second incision for artificial anus was made, in the left inguinal region. When the peritoneum was incised, about six ounces of straw-colored fluid escaped; and the first thought was that the bladder had been perforated. Digital examination, however, proved it to be the abdominal cavity. The intestines were entirely collapsed, and it was with some difficulty that a sling of intestine was secured and fastened with sutures in the wound, and when done, it was not certain whether he had to do with the colon or small intestine.

When the intestine was incised, a small quantity of gas escaped, but no meconium or fæces. The wound was dressed with charpie, and the child sent home breathing somewhat more regularly and less cyanotic. Two days afterwards, the child died, and *post mortem* examination demonstrated extensive peritonitis, with the rectum terminating in an oval sac, near the neck of the uterus.

Operations for artificial anus are sometimes made in the lumbar region, where an incision of the peritoneum is unnecessary; but in case of collapse of the intestines, as in the above case, it would be much more difficult to secure a portion of the bowel, on account of the consistence of the parts, than would be the case in the inguinal region.

The Professor said, it was not ordinarily advisable to operate in such cases, until the child was 24 hours old, or more, as then the rectum would be more likely to be distended with meconium, and, consequently, more probability of its being reached. I think he said he had operated three or four times in the lumbar region, but without success.

Professor Braun has gone to Rome, I understand with the Empress Elizabeth, to be present at the accouchment of her sister, the Ex-Queen of Naples. His assistant, Dr. Mayerhofer, lectures during his absence.

A boy, 12 years old, with epispadias or deficiency of the anterior wall of the bladder, in much the same condition, I think, as a young man in the United States, who goes about exhibiting himself to medical schools and hospitals, was admitted to the hospital here, in Oct.; and on the 25th of the same month, underwent a plastic operation to supply the deficiency.

An oval flap, four or five inches long, was taken from the region of the umbilicus and turned down, so that the cutaneous portion of the flap now formed the internal wall of the bladder, and was attached to the prepared edges by several sutures For some days after the operation, it was difficult to tell whether the whole flap had necrosed or not, except by trying the sensation and life, by the prick of a needle.

*Nov. 3d.* The necrosed portions of the flap were removed, but it had made no union, and its edges were entirely free, though it was somewhat curled up. The surface from which the flap was taken is covered with healthy granulations and healing over.

*Nov. 11th.* The flap was again brought down, its edges pared and attached by wire sutures, enough of the flap still remaining to form the anterior wall.

*Nov. 15th.* The flap had united in several places, but last night the patient had the nightmare, sprung up in bed, and separated all the adhesions. The flap is now too small to supply the deficiency and has a tendency to roll back from whence it was cut.

*Nov. 25th.* A cicatrix covers nearly one-half of the surface from which the flap was taken. The edges of the flap are cicatrizing, which is now comparatively small and stands up perpendicular to the abdomen.

The general health remains good.

*Dec. 5th.* The case remains about the same as at last date. Another operation will soon be made, taking a flap from one of the inguinal regions. If that does not succeed, another will be taken from the other inguinal region, and, finally, if necessary, the scrotum will be used. Should none of these succeed, I presume the case will be abandoned.

Will furnish further notes of the case.

Yours truly, F.

